# Supplementary material for: A Critical View of the Application of the APEX Software (Aqueous Photochemistry of Environmentally-Occurring Xenobiotics) to Predict Photoreaction Kinetics in Surface Freshwaters
Source: Molecules. 2019 Dec 18;25(1):9. doi: 10.3390/molecules25010009 (PMC7017383; doi:10.3390/molecules25010009)
Supplement: Supplementary file 1 [file molecules-25-00009-s001.zip › 3.2.4_gcc-4.4.0.zip!/3.2.4_gcc-4.4.0/3.2.4_gcc-4.4.0/doc/octave/html/interpreter/Administrating-Packages.html]

Administrating Packages - Untitled


Next: Creating Packages,
Previous: Using Packages,
Up: Packages


---

### 35.3 Administrating Packages

On UNIX-like systems it is possible to make both per-user and
system-wide installations of a package. If the user performing the
installation is `root` the packages will be installed in a
system-wide directory that defaults to
OCTAVE\_HOME/share/octave/packages/. If the user is not
`root` the default installation directory is
~/octave/. Packages will be installed in a subdirectory of the
installation directory that will be named after the package. It is
possible to change the installation directory by using the
`pkg prefix` command

```
     pkg prefix new_installation_directory
```

The current installation directory can be retrieved by typing

```
     current_installation_directory = pkg prefix
```

To function properly the package manager needs to keep some
information about the installed packages. For per-user packages this
information is by default stored in the file ~/.octave\_packages
and for system-wide installations it is stored in
OCTAVE\_HOME/share/octave/octave\_packages. The path to the
per-user file can be changed with the `pkg local_list` command

```
     pkg local_list /path/to/new_file
```

For system-wide installations this can be changed in the same way
using the `pkg global_list` command. If these commands are
called without a new path, the current path will be returned.
